# Kinetic studies and homology modeling of a dual-substrate linalool/nerolidol synthase from *Plectranthus amboinicus*

**DOI:** 10.1038/s41598-021-96524-z

**Published:** 2021-08-24

**Authors:** Nur Suhanawati Ashaari, Mohd Hairul Ab. Rahim, Suriana Sabri, Kok Song Lai, Adelene Ai-Lian Song, Raha Abdul Rahim, Janna Ong Abdullah

**Affiliations:** 1 Department of Cell and Molecular Biology, Faculty of Biotechnology and Biomolecular Sciences, Universiti Putra Malaysia, 43400 UPM Serdang, Selangor, Malaysia; 2 Department of Industrial Biotechnology, Faculty of Industrial Sciences and Technology, Universiti Malaysia Pahang, 26300 Gambang, Kuantan, Pahang Malaysia; 3 Enzyme and Microbial Technology Research Center, Faculty of Biotechnology and Biomolecular Sciences, Universiti Putra Malaysia, 43400 UPM Serdang, Selangor, Malaysia; 4 Department of Microbiology, Faculty of Biotechnology and Biomolecular Sciences, Universiti Putra Malaysia, 43400 UPM Serdang, Selangor, Malaysia; 5 Health Sciences Division, Abu Dhabi Women’s College, Higher Colleges of Technology, 41012 Abu Dhabi, United Arab Emirates

**Keywords:** Biochemistry, Computational biology and bioinformatics, Molecular biology

## Abstract

Linalool and nerolidol are terpene alcohols that occur naturally in many aromatic plants and are commonly used in food and cosmetic industries as flavors and fragrances. In plants, linalool and nerolidol are biosynthesized as a result of respective linalool synthase and nerolidol synthase, or a single linalool/nerolidol synthase. In our previous work, we have isolated a linalool/nerolidol synthase (designated as *PamTps1*) from a local herbal plant, *Plectranthus amboinicus*, and successfully demonstrated the production of linalool and nerolidol in an *Escherichia coli* system. In this work, the biochemical properties of *Pam*Tps1 were analyzed, and its 3D homology model with the docking positions of its substrates, geranyl pyrophosphate (C_10_) and farnesyl pyrophosphate (C_15_) in the active site were constructed. *Pam*Tps1 exhibited the highest enzymatic activity at an optimal pH and temperature of 6.5 and 30 °C, respectively, and in the presence of 20 mM magnesium as a cofactor. The Michaelis–Menten constant (*K*_m_) and catalytic efficiency (*k*_*cat*_/*K*_m_) values of 16.72 ± 1.32 µM and 9.57 × 10^–3^ µM^−1^ s^−1^, respectively, showed that *Pam*Tps1 had a higher binding affinity and specificity for GPP instead of FPP as expected for a monoterpene synthase. The *Pam*Tps1 exhibits feature of a class I terpene synthase fold that made up of α-helices architecture with N-terminal domain and catalytic C-terminal domain. Nine aromatic residues (W268, Y272, Y299, F371, Y378, Y379, F447, Y517 and Y523) outlined the hydrophobic walls of the active site cavity, whilst residues from the RRx_8_W motif, RxR motif, H-α1 and J-K loops formed the active site lid that shielded the highly reactive carbocationic intermediates from the solvents. The dual substrates use by *Pam*Tps1 was hypothesized to be possible due to the architecture and residues lining the catalytic site that can accommodate larger substrate (FPP) as demonstrated by the protein modelling and docking analysis. This model serves as a first glimpse into the structural insights of the *Pam*Tps1 catalytic active site as a multi-substrate linalool/nerolidol synthase.

## Introduction

Terpenoids are structurally diverse and are the most abundant natural products among the myriad of compounds produced by plants, with biological roles ranging from growth and development to intracellular signaling and defense against predatory species^1^. Applications of these valuable compounds in the industries include as pharmaceuticals, flavors, fragrances and biofuels^2^. In higher plants, terpenoids are synthesized *via* either the cytosolic mevalonate (MVA) pathway or the plastidial methylerythritol phosphate (MEP) pathway, where the precursors are converted into structurally diverse terpenoids by the family of terpene synthases (TPSs). Sesquiterpene synthases responsible for sesquiterpenes (C_15_) production are localized in the cytosol, whereas monoterpene synthases that catalyze the production of monoterpenes (C_10_) are present in the plastids. Monoterpene synthases (600–650 amino acids) are longer than sesquiterpene synthases (550–580 amino acids) due to their N-terminal signal peptides that target the initial translation products towards the plastid^3^. A number of plant monoterpene and sesquiterpene synthases of molecular masses ranging from 50 to 100 kDa (monomers or homodimers) have been isolated and characterized with similar properties such as requirement for a divalent metal ion, having pI value near 5.0 and pH optimum within a unit of neutrality^4^.

Despite the lack of significant sequence similarities, terpene synthases share highly conserved tertiary and quaternary structural features dominated by α-helical folds known as class I terpene synthase fold^5,6^. These proteins consist entirely of α-helices and short connecting loops and turns that are organized into two structural domains of a non-functional N-terminus and a catalytically active C-terminus^7^. The class I terpene synthases which include monoterpene and sesquiterpene synthases utilize a trinuclear magnesium cluster coordinated by two conserved metal-binding motifs (DDxxD and NSE/DTE) to initiate catalysis^8^. The trinuclear magnesium cluster facilitates orientation of the substrate diphosphate moiety in the active site and triggers substrate ionization that generates reactive carbocation intermediates which undergo a series of cyclization, hydride shifts or other arrangements until the reactions are terminated by protons loss or by the addition of water^7,9^. The ligand binding causes conformational changes that cap and sequester the active site, thereby protecting the reactive carbocation intermediates from premature quenching by bulk solvents^5,8^.

One of the most fascinating features of the terpene synthases group is its ability to form a single product or multiple products from a sole substrate^4,7,10,11^. Furthermore, some terpene synthases exhibit multi-substrate abilities by synthesizing terpenes of different chain lengths depending on the corresponding substrate availability^12–15^. The structural basis of fidelity and promiscuity of the terpene synthases is related to the contour of the active site that serves as a template for catalysis by ensuring substrates and intermediates bind in the proper conformations, thereby controlling the formation of final catalysis product(s)^16–18^. Accordingly, the active site contours are product-like especially for high fidelity synthases to ensure the generation of specific product(s)^8^.

Linalool/nerolidol synthase is a multi-substrate enzyme with the capability to use GPP or FPP as a substrate, leading to the synthesis of linalool or nerolidol, respectively. Linalool participates in a complex interplay between pollinator attraction and plant defense against herbivory by attracting natural enemies of the herbivores^19,20^. Similarly, nerolidol has been identified as a potent signal that induces accumulation of defense-related compounds with extensive natural anti-herbivore or anti-pathogen effects^21,22^. These compounds are widely used as fragrance materials in cosmetic products including perfumes, lotions and shampoos, and in non-cosmetic products such as detergents and cleansers. Isolation and characterization of this enzyme were reported from *Plectranthus amboinicus*^15^, *Rosa chinensis*^14^, *Hedychium coronarium*^23^, *Vitis vinifera*^24^ and *Antirrhinum majus*^13^ which showed that this type of bifunctional enzyme is widespread across multiple plant species. The multi-substrate activity may confer advantages on plants to adapt rapidly in response to changes in the substrate profile under perturbation of metabolism in stressed plants, as well as under certain developmental changes without compromising their central metabolism^12^.

In our previous study, a putative monoterpene synthase gene (*PamTps1*) was isolated from *P. amboinicus* and introduced into the *E. coli* Rosetta™ 2 (DE3), which resulted in the production of linalool and nerolidol. Functional characterization demonstrated that this multi-substrate enzyme predominantly catalyzed formation of linalool and nerolidol from GPP and FPP, respectively, and was designated as a linalool/nerolidol synthase (Accession no: QGN03393)^15^. To learn more about *Pam*Tps1, biochemical characterization such as pH dependence, temperature dependence, divalent metal ion and substrate preferences, and kinetic properties were investigated. A reliable 3D homology model depicting the conformation of the *P. amboinicus* linalool/nerolidol synthase and the position of both GPP and the FPP substrates in the active site were also predicted in this analysis. Identification of the key residues involved in the active site architecture and catalysis reaction were also conducted. This model will serve as a basis for protein engineering to improve this bifunctional synthase with regard to product specificity or catalytic efficiency, and as a guide to future exploitations of this enzyme in terpenoids production.

## Results and discussion

### Effects of pH and temperature on *Pam*Tps1 activity

**Figure 1:**
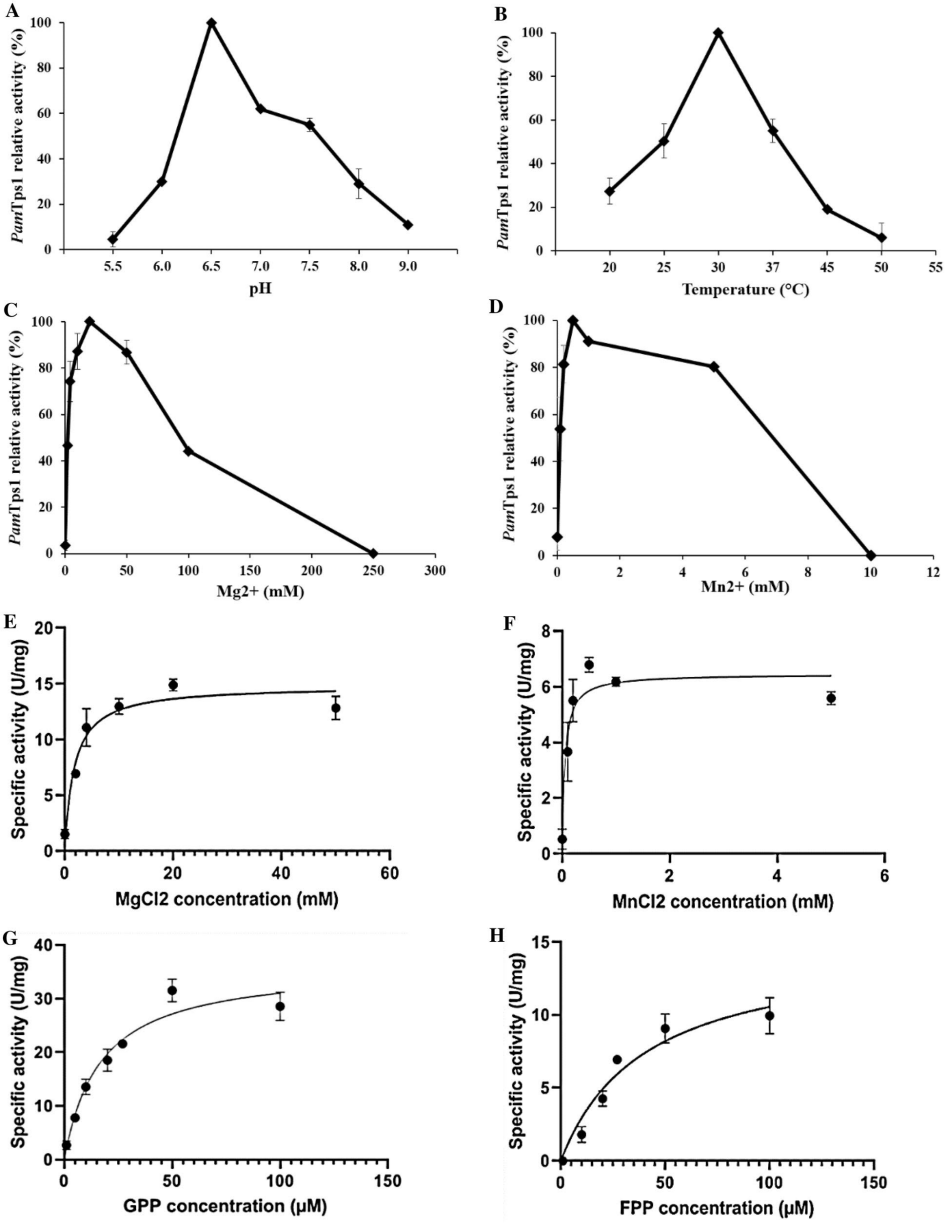
Biochemical characterization of *Pam*Tps1. (**A**) pH; (**B**) Temperature; (**C**) Mg^2+^ concentrations; (**D**) Mn^2+^ concentrations. Michaelis–Menten plot of *Pam*Tps1 at different concentrations of (**E**) Mn^2+^; (**F**) Mg^2+^; (**G**) GPP and (**H**) FPP. The saturation curve was constructed using Michaelis–Menten equation by hyperbolic regression. Values were reported as the mean of relative activity ± SD of triplicate analysis.

The *Pam*Tps1 activity was investigated using GPP as a substrate over a pH range of 5.5 to 9.0. At pH 6.5, the maximum catalytic activity was observed but was reduced to less than 10% of the maximum activity at pH 5.5 and pH 9 (Fig. 1A). This result was similar to the *3R*-linalool synthase of *Mentha citrate* which exhibited an optimum pH close to pH 6.5 and a half maximum velocity at pH 7.5^25^. Typically, the optimal pH for terpene synthases is within a unit of neutrality as reviewed by Bohlmann et al.^4^. Previously characterized plant linalool synthases showed an optimal pH range of 6.0–8.0^23,26–29^. It was also noted that monoterpene synthases had a pH optima of 6–7 that correlated with the pH of the chloroplast in plants^30–32^, which corroborated the findings of *Pam*Tps1. Solvolytic decomposition of GPP to linalool in the presence of divalent cation was reported to occur under acidic condition^33^. As a result, the effect of pH below 5.5 could not be determined accurately due to an increase of substrate decomposition to linalool, which was also observed by Crowell et al.^25^.

The enzymatic activity of *Pam*Tps1 was conducted at temperatures ranging from 25 to 50 °C. Optimal catalytic activity was observed at 30 °C, with only half of the maximal activity noted at 25 °C and 37 °C (Fig. 1B). The observed result was similar to the temperature range (30–40 °C) reported for plant terpene synthases such as ocimene synthase of *Lilium*^26^, linalool synthase of coriander^34^ and *Hedychium coronarium*^23^, cineole synthase of lavender^35^ and β-sesquiphellandrane synthase of *Persicaria minor*^36^. The catalytic activity of *Pam*Tps1 dropped drastically beyond the optimum temperature, with only less than 10% of the full velocity retained at 50 °C. This could probably be linked to the destabilization of the three-dimensional structure of the enzyme at higher temperatures and ultimately contributed to denaturation and irreversible loss of activity^37^.

### Effects of divalent metals on *Pam*Tps1 activity

Terpene synthases have an absolute requirement for a divalent metal ion such as Mg^2+^ or Mn^2+^ as a cofactor. The role of divalent metal ion in terpene synthases catalysis has been widely discussed and presumably involved in both substrate binding and catalysis^8,17^. Chelation of metal ion such as Mg^2+^, neutralizes two of the three negative charges of the diphosphate moiety of the substrate, thereby assisting the ionization of the allylic substrate into highly reactive carbocation intermediates^38^. Thus, divalent metal ions preferences of *Pam*Tps1 and their influence on the catalytic activity were evaluated at different concentrations of Mg^2+^ (0–250 mM) and Mn^2+^ (0–10 mM).

In the absence of a divalent metal ion, the *Pam*Tps1 activity was negligent. However, the activity was restored by the provision of either Mg^2+^ or Mn^2+^, which suggested an absolute requirement for a metal ion cofactor for catalytic activity (Fig. 1C,D). A maximal activity was obtained with Mn^2+^ at 0.5 mM, but was inhibited as Mn^2+^ concentration increased to 10 mM (Fig. 1D). Other characterized plant terpene synthases demonstrated maximum activity with manganese concentrations at less than 1.0 mM^28,39,40^. On the other hand, in the presence of Mg^2+^, the catalytic activity of *Pam*Tps1 increased steadily from 2 mM to a maximum activity at 20 mM, but was inhibited at 250 mM (Fig. 1C). This optimal concentration of Mg^2+^ finding was also observed in *M. citrata* linalool synthase^25^, *Citrus sinensis* limonene synthase^40^ and Japanese pepper terpene synthases^39^. In this study, *Pam*Tps1 showed a preference for Mg^2+^ for catalysis with 2.1 folds increase in activity compared to Mn^2+^. Likewise, other characterized plant terpene synthases that favored Mg^2+^ over Mn^2+^ included linalool/nerolidol synthase 1 and 2^13^, *Artemisia annua* monoterpene synthases^41^, *Lilium* ‘Siberia’ terpene synthase^26^ and *Santalum album* terpene synthases^42^. In contrast, linalool synthase of lavender^28^ and *C. sinensis* limonene synthase^40^ showed preferences for Mn^2+^ as a cofactor with high terpene yields when 1–5 mM of Mn^2+^ were used.

### Kinetic parameters of *Pam*Tps1

In this study, *Pam*Tps1 activity was inhibited when Mg^2+^ and Mn^2+^ concentrations beyond 50 mM and 5 mM, respectively, were used. Therefore, the *K*_m_ value was estimated by a non-linear Michaelis–Menten curve using lower concentrations of Mg^2+^ and Mn^2+^ (Fig. 1E,F) which gave 1.74 ± 0.35 mM and 0.05 ± 0.001 mM, respectively, (Table 1). These values were comparable to those obtained with kiwi terpene synthases^43^, snapdragon linalool/nerolidol synthase^13^ and sweet basil geraniol synthase^44^. Nevertheless, in some reported metal ions studies, there are other terpene synthases that recorded *K*_m_ values of less than 1 mM^23,27,45^ while higher *K*_m_ values were also noted in some terpene synthases including *A. annua* linalool synthase^46^ and γ-terpinene synthases^10,47^. Although *K*_m_ value for *Pam*Tps1 was substantially lower when using Mn^2+^, its *V*_*max*_ value was only 43% of that with Mg^2+^. It is presumed that *PamT*ps1 is more likely to operate with Mg^2+^ cofactor in planta due to the higher concentration of Mg^2+^ in plant cells as compared to the Mn^2+^ ^48,49^.

**Table 1:** Kinetic properties of *Pam*Tps1.

Metal/substrate	*V*_*max*_ (µmol mg^−1^)	*K*_m_	*k*_*cat*_ (s^−1^)	*k*_*cat*_/*K*_m_
Mg^2+^*	14.84 ± 1.40	1.74 ± 0.35 mM	0.10	0.058 mM^−1^ s^−1^
Mn^2+^*	6.46 ± 0.35	0.05 ± 0.00 mM	0.04	0.800 mM^−1^ s^−1^
GPP**	24.16 ± 3.75	16.72 ± 1.32 µM	0.16	9.57 × 10^–3^ µM^−1^ s^−1^
FPP**	14.85 ± 2.80	40.47 ± 3.83 µM	0.10	2.47 × 10^–3^ µM^−1^ s^−1^

*Values when Mg^2+^ and Mn^2+^ were used in the presence of 27 µM GPP.

**Values for GPP and FPP were measured in the presence of 20 mM Mg^2+^.

*V*_*max*_ = Maximal velocity; *K*_m_ = Michaelis–Menten constant; *k*_*cat*_ = Turnover number; *k*_*cat*_/*K*_m_ = Catalytic efficiency.

Values were reported as the mean of relative activity ± SD of triplicate analysis.

Kinetic characterization of *Pam*Tps1 for GPP and FPP was performed below 100 µM since higher concentrations inhibited its catalytic activity (Fig. 1G,H). The apparent *K*_m_ value of *Pam*Tps1 for GPP was 16.72 ± 1.32 µM, which was well within the range of *K*_m_ values reported in other plant monoterpene synthases (Table 1)^40,41^, but lower compared to linalool synthases from *H. coronarium* (20.54 ± 4.52 µM)^23^, *Cinnamomum osmophloeum* (54.19 µM)^50^, *L. angustifolia* (55.8 ± 4.1 µM)^28^ and *M. citrata* (25 ± 6.00 µM)^25^. Nonetheless, linalool synthases from snapdragon^13^, *A. arguta*^27^ and *A. chinensis*^51^ exhibited *K*_m_ values below 10 µM which suggested that these enzymes have a higher affinity for GPP. On the other hand, *Pam*Tps1 *K*_m_ value of 40.47 ± 3.83 µM for FPP was 2.4 folds higher than that for GPP, which signified that *Pam*Tps1 had a higher binding affinity for GPP.

From the abovementioned results, it can be inferred that *Pam*Tps1 has a lower affinity for FPP and become saturated at a higher substrate concentration to reach its maximal velocity (*V*_*max*_ = 14.85 ± 2.80 µmol mg^−1^). *Pam*Tps1 has a greater affinity for GPP than FPP as anticipated for a monoterpene synthase, where a lower concentration of GPP was required to achieve *V*_*max*_ of 24.16 ± 3.75 µmol mg^−1^. Similar observation was noted in the snapdragon linalool/nerolidol synthases that exhibited higher substrate affinity towards GPP than to FPP^13^. The turnover rate (*k*_*cat*_) for both substrates in the current study were 0.16 s^−1^ and 0.10 s^−1^ for GPP and FPP, respectively, which was within the range of monoterpene (0.01–1.0 s^−1^) and sesquiterpene (0.03–0.5 s^−1^) synthases recorded^52,53^, and the low *k*_*cat*_ values reflected that *Pam*Tps1 is a relatively slow enzyme. Terpene synthases are typically slow enzymes, which is a general feature of the enzymes involved in secondary metabolism and is approximately 30 folds slower than those involved in central metabolism^54^. The catalytic efficiency (*k*_*cat*_/*K*_m_) of GPP was 3.9 folds higher than FPP, further suggesting that *Pam*Tps1 recognized GPP more efficiently, which was in accordance with the abovementioned expectations. This may also be linked to the fact that *Pam*Tps1 was a plastid-targeted enzyme, where the GPP pool was located. Parallel observations were seen in lavender^28^ and *Freesia*^55^.

### Secondary structure prediction

The secondary structure of *Pam*Tps1 was predicted using PSIPRED server^56^ followed by identification and annotation of the protein domain using MOTIF and SMART^57^. The PSIPRED tool predicted that the secondary structure of *Pam*Tps1 would consist entirely of α-helices (24 α-helices) connected by coils, with no strands or β-sheets observed except for the two extended strands located at the N-terminal signal peptide region (Fig. S1). Through domain analysis, it was revealed that these α-helices were organized into two structural domains of N-terminal (residues: 66–245) (Pfam: PF01397) and C-terminal metal binding domain (residues: 277–540) (Pfam: PF03936) with domain boundary located at residue M271 as determined by DomPRED. These predictions are in agreement with general features of most plant terpene synthases that adopt an α-helical architecture, which are organized into two domains of N-terminal region that has structural similarity to glycosylhydrolases^58^ and the C-terminal domain containing the catalytic site^5^.

### Protein homology modelling of the *Pam*Tps1

**Figure 2:**
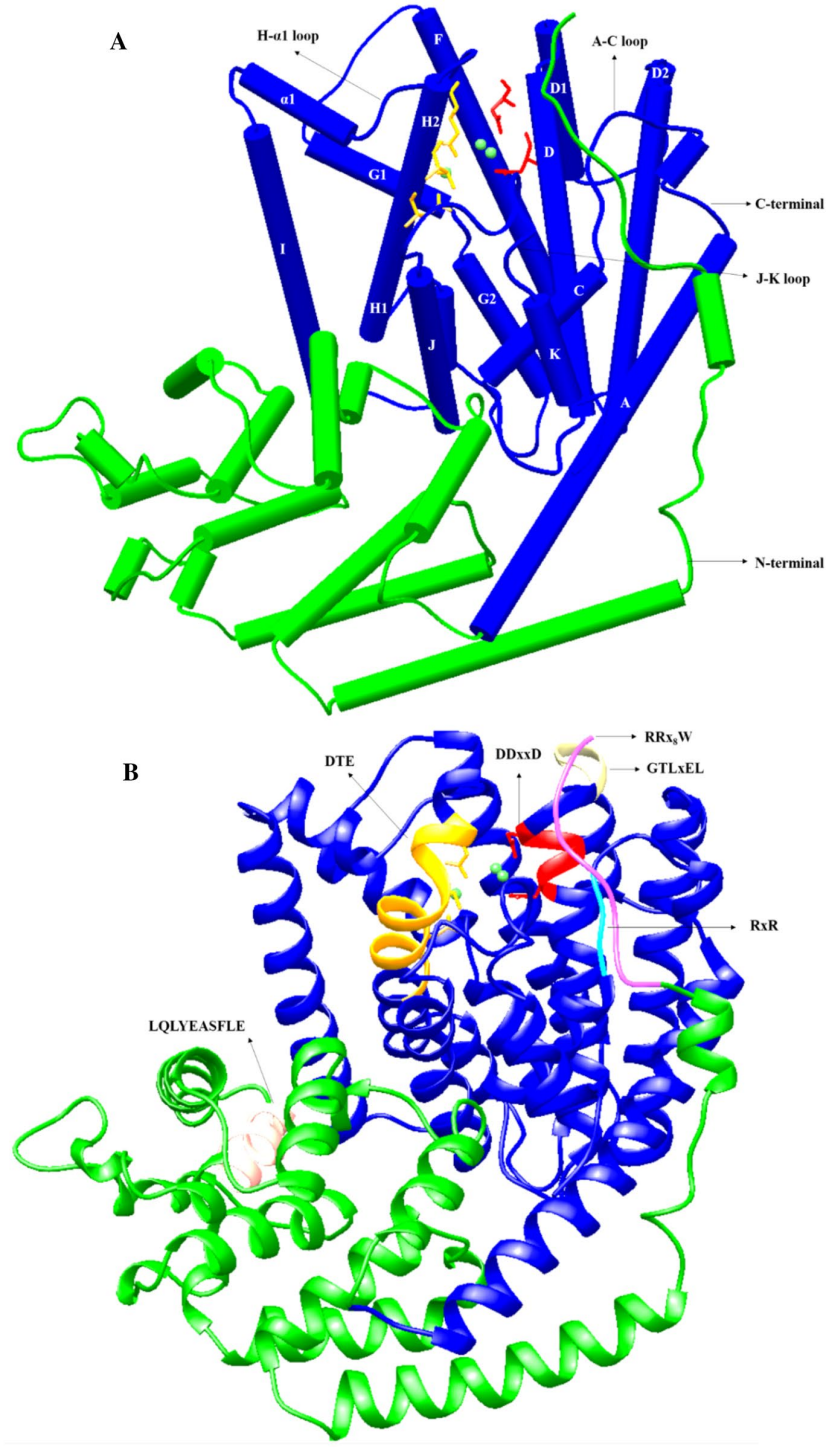
Protein homology modelling of *Pam*Tps1 using SWISS-MODEL server showed the structural domains and the active site of the enzyme. (**A**) model structure of *Pam*Tps1 made up of α-helices with N-terminal domain (green) and C-terminal domain (blue). (**B**) Ribbon view of *Pam*Tps1 model. The helical segment was designated according to Tarshis et al.^67^. All conserved motifs were labelled in the figure and Mg^2+^ was illustrated as green spheres.

The *Pam*Tps1 was modeled on the crystal structure of *Salvia officinalis* (+)-bornyl diphosphate synthase (BPPS) (1N24)^5^ using residues that correspond to the complete amino acid sequence in accordance to the RRx_8_W motif. The chosen BPPS template featured a closed active site conformation with Mg^2+^ and its product, and shared 67.04% sequence identity. The residue numbers described hereafter corresponded to the numbering of amino acids immediately following the RRx_8_W motif (Fig. S2). The predicted *Pam*Tps1 structure as shown in Fig. 2, revealed that the enzyme comprised of two structural domains of N- and C-terminal, connecting with short loops and turns. The N-terminal domain (residues 1–214) of *Pam*Tps1 consisted of 14 α-helices arranged in an α-barrel with minor structural differences to that of BPPS^5^. Although there was no established catalytic function for this N-terminal domain, it was reported that this domain was involved in capping the active site pocket upon substrate binding, and presumably shielded the reactive carbocation intermediates from water as observed in the crystal structure of BPPS, *Taxus brevifolia* taxadiene synthase (PDB ID: 3P5R) and *Gossypium arboretum* δ-cadinene synthase (PDB ID: 3G4F)^5,9,59^. The presence of this apparently non-functional N-terminal domain in terpene synthases may have been due to an evolutionary vestige from copalyl diphosphate synthase-kaurene synthase, which was the ancestor of all modern terpene synthases that possess both functional catalytic domains^6,8,60^.

The N-terminal domain contained two conserved motifs that were present in typical plant terpene synthases, namely the RRx_8_W and LQLYEASFLL motifs. The tandem arginine motif was found in many plant monoterpene synthases and was thought to mark the approximate cleavage site of the plastid-targeting sequence^6^. A previous truncation study of this motif from a limonene synthase suggested that the RR motif was required for initial isomerization of GPP to linalyl diphosphate (LPP), owing to the inability of the truncated limonene synthase to accept GPP as a substrate, while still functioning with LPP as a substrate for the cyclization step^6,61^. These arginine residues may also contribute to the stabilization of the closed active site while still allowing flexibility that was necessary for the binding of two structurally different prenyl diphosphates (GPP and LPP) as observed in limonene synthase^6^. Since *Pam*Tps1 did not undergo a cyclisation reaction, it was likely that the RRx_8_W motif might only be involved in the capping of the *Pam*Tps1 active site and not in the catalysis reaction. The InterProScan analysis also predicted that the RRx_8_W region acted as an active site lid in the *Pam*Tps1. Besides that, the LQLYEASFLL motif that was assumed to be part of the active site^62,63^ occurred as LQLYEASFLE in *Pam*Tps1, and there were no observable differences in the overall structure of the enzyme for amino acid substitution from leucine to glutamic acid.

The larger C-terminal domain (residues 215–542) adopted an α-helical architecture known as class I terpene synthase fold which consisted of 16 α-helices, where the hydrophobic pocket of the active site cavity was formed by six α-helices (C, D, F, G, H and J) (Fig. 2). This domain was well conserved with an RMSD value of 0.190 Å as compared to the BPPS. The C-terminal domain contained two metal binding motifs of the aspartate-rich DDxxD and NSE/DTE (evolved from a second aspartate-rich region) to form a consensus sequence of (L,V)(V,L,A)(**N**,**D**)D(L,I,V)x(S,**T**)xxx**E**. The NSE/DTE motif appeared to be less well conserved amongst the plant terpene synthases as compared to the DDxxD motif. Both the DDxxD and NSE/DTE motifs were reported to bind to a trinuclear magnesium cluster involved in the fixation of the diphosphate substrate^5,64,65^. The *Pam*Tps1 also contained other motifs that were thought to be part of the terpene synthases active site, such as RxR and GTLxEL^63,66^ which occurred as RDR and GTLDEL in *Pam*Tps1 and were located 35 amino acids upstream and two amino acids downstream of the DDxxD, respectively.

**Figure 3:**
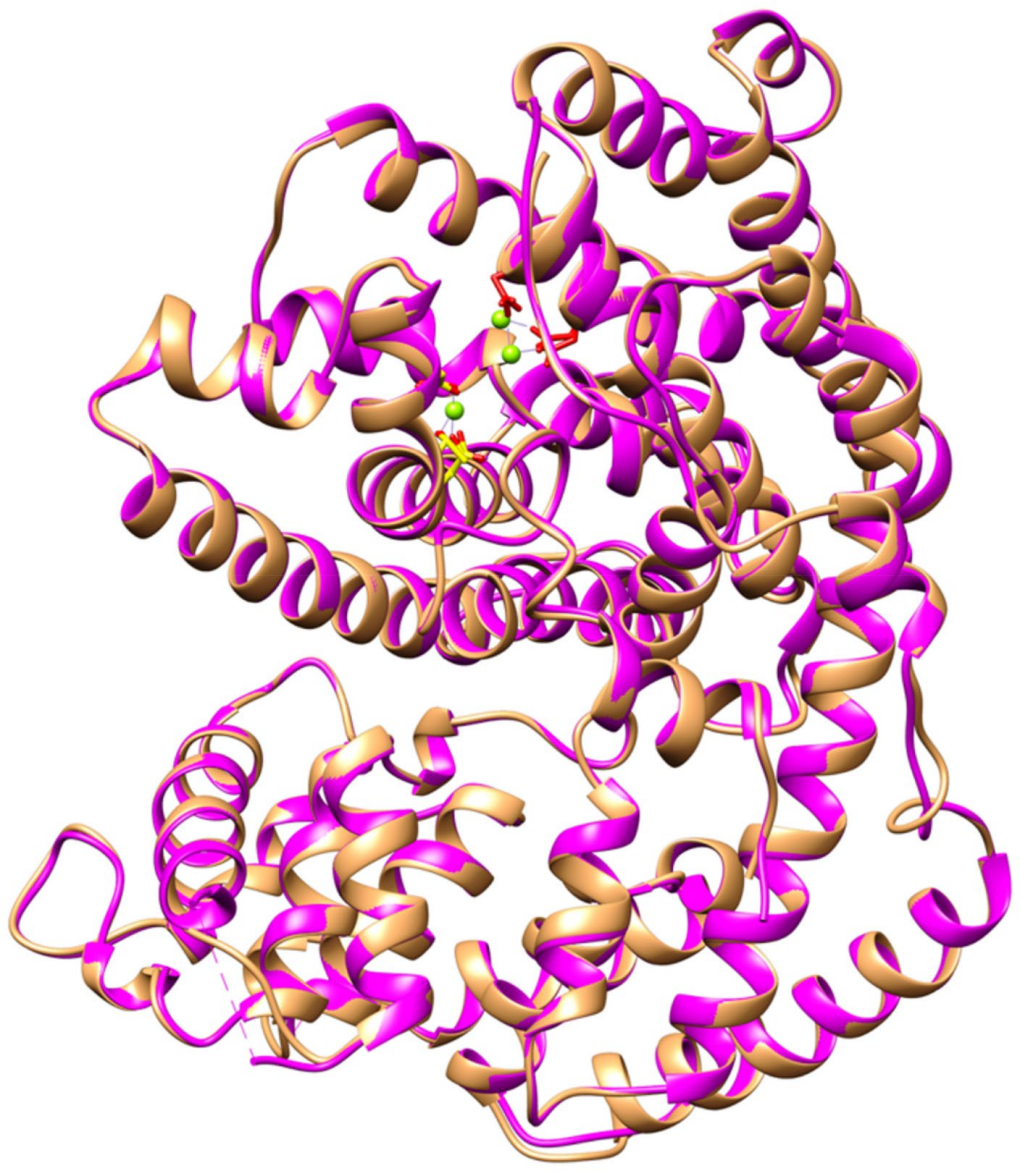
Superimposition of *Pam*Tps1 model (purple) with BPPS template (brown) using Chimera. The α-carbon RMSD value of 0.203 Å indicated the two structures were exceptionally similar. The aspartate-rich motif was red, the DTE motif was orange and the green spheres were magnesium ions.

Protein structural alignment or superimposition allows homology establishment between template and protein model based on the 3D protein conformation as a protein structure was more conserved than its sequence during evolution. Superimposition of the *Pam*Tps1 model with BPPS template using Chimera with α-carbon RMSD fitted to 0.203 Å showed that the two structures were exceptionally similar (Fig. 3).

### Validation of the *Pam*Tps1 model

The reliability of the model was first evaluated by the GMQE and Qualitative Model Energy Analysis (QMEAN) scores provided by the SWISS-MODEL tool. The GMQE score is expressed as a number between 0 and 1, where higher numbers indicate higher reliability of the model^68^. The QMEAN Z-score provides an estimate degree of structural features similarity observed in the model with scores around 0 indicate good agreement between model structure and template^69^. The *Pam*Tps1 model scores of 0.82 and − 1.32 for respective GMQE and QMEAN showed that the built model was reliable and satisfactory. Further validation by PROCHECK to assess the stereochemical quality of generated model showed that 92.8% of *Pam*Tps1 residues fall in most favored regions, 6.6% residues in additional allowed regions, 0.2% residues in generously allowed regions and only 0.4% residues in the disallowed regions suggesting the acceptability of the modeled structure (Fig. S2, Table S3). PROVE analysis revealed that the quality of the predicted 3D structure of *Pam*Tps1 model was good and reliable with the respective Z-score mean and Z-score RMS for the entire structure of 0.487 and 1.421, respectively. The ERRAT analyses statistic of non-bonded interactions between different atom types based on characteristic atomic interactions^97^. The overall ERRAT quality factor value is expressed as the percentage of the protein for which the calculated value is less than the 95% rejection limit. A good high-resolution structure typically yields values of 95% or higher, and the *Pam*Tps1 model yielded an overall quality factor of 95.88%, which was very satisfactory. Another program used for validation of protein structure was the Verify3D, which determines compatibility of an atomic model (3D) with its own amino acid sequence (1D) by assigning structural class based on its location and environment^98^. The Verify3D analysis of *Pam*Tps1 model revealed that 95.73% of the residues had an average 3D–1D score ≥ 0.2. As the cut-off score ≥ 0, this implies that the predicted model was valid. ProSA was used to check the 3D model of *Pam*Tps1 for potential errors where positive value of the z-score corresponded to problematic or erroneous region of a model. The Z-score of − 12 for *Pam*Tps1 model was within the acceptable range of X-ray studies and this value was close to the value of the template (− 10.92) suggesting that the predicted model was reliable and close enough to experimentally determine structure (Fig. S2, Table S3).

### Molecular docking of *Pam*Tps1 with prenyl diphosphate substrates

To gain further insight into the active site of the enzyme investigated here, the model structure of *Pam*Tps1 was carried out with molecular docking using GPP (C_10_) and FPP (C_15_) substrates. Docking of the prenyl diphosphate substrates yielded multiple docking positions. The criteria for choosing the best docking position were based on the lowest docking score and the number of hydrogen bonds between the substrate and the amino acid residues. A docking position with the least docking score has the highest affinity towards the ligand, and hence is the best docked conformation. Hydrogen bonds contribute to the stability of proteins and specificity of protein–ligand interactions, which is also an important consideration for selection of the docking position^70^. The docking results were further analyzed using Chimera and LigPlot + to generate 2D and 3D ligand–protein interaction diagrams, respectively.

**Figure 4:**
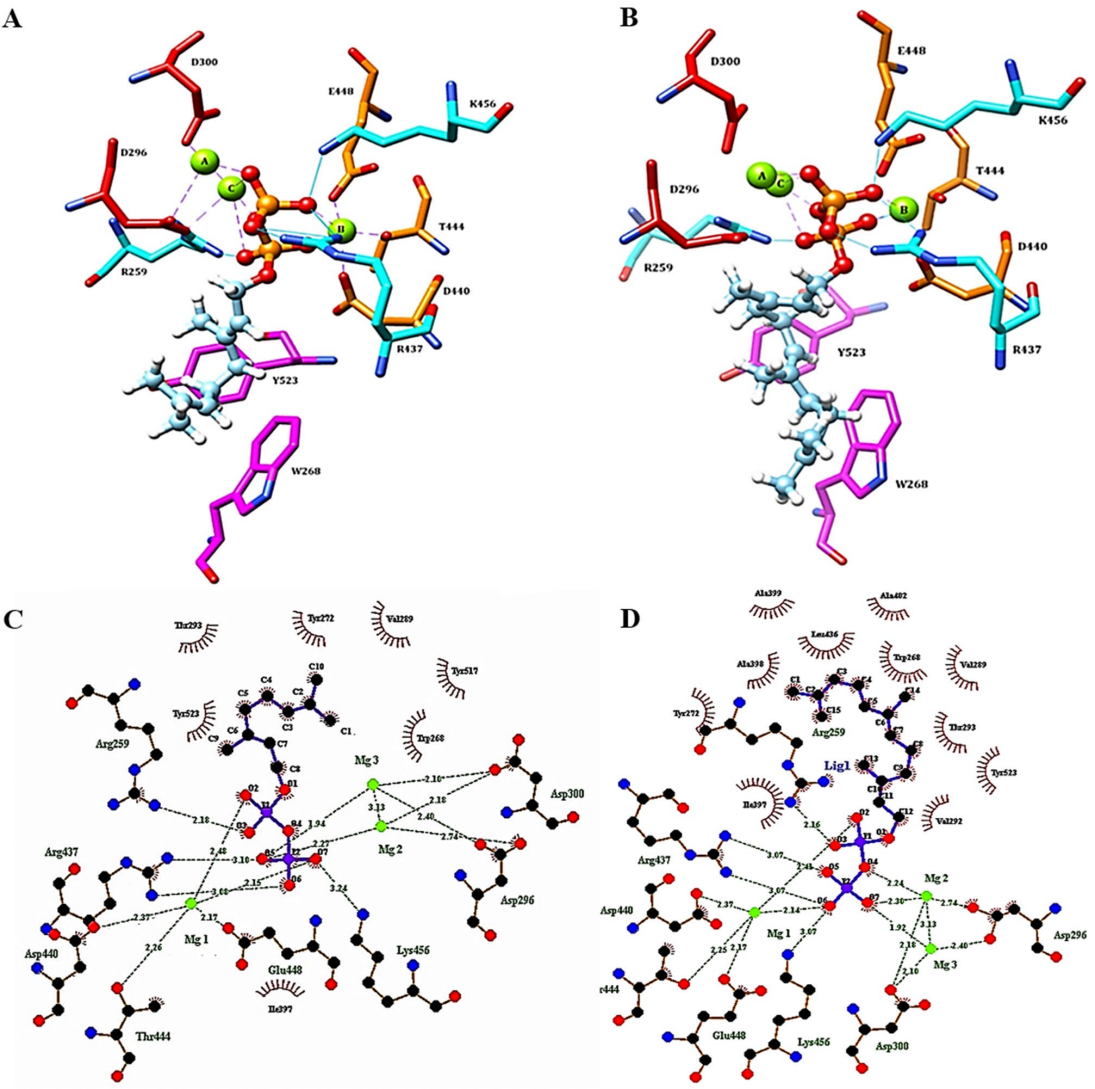
Ligand-*Pam*Tps1 interactions. Three-dimensional (3D) view of (**A**) GPP and (**B**) FPP docking at *Pam*Tps1 active site. The red–orange balls and stick chains represent PPi, green spheres are the Mg^2+^, the red side chains are the DDxxD motif, the orange side chains are the DTE motifs, the blue side chains are the hydrogen bond donor residues, the magenta side chains represent the aromatic residues and the cyan lines are the hydrogen bonds. Two-dimensional (2D) view of (**C**) GPP and (**D**) FPP docking at *Pam*Tps1 active site. Hydrogen bonds are shown as green dotted lines, Mg^2+^ are shown in green spheres and spoked arcs represent residues making non-bonded contacts with the hydrophobic tail of the ligand. Details of the docking result are summarized in Table S2.

Docking of GPP and FPP substrates confirmed that the active site of *Pam*Tps1 was located at the C-terminal domain, proximate to the location of the Mg^2+^ cofactor (Fig. 4). A two-dimensional representation of Mg^2+^ interaction with the amino acid residues and substrate (ligand) was displayed in Fig. 4C,D. This concurred with earlier observations using SWISS-MODEL and InterProscan that the diphosphate (PPi) moiety of the prenyl substrates interacted with the highly conserved aspartate-rich (**D**296DVY**D**300) and NSE/DTE (LA**D**440DLG**T**444APF**E**448) motifs via complexed Mg^2+^, in which the boldface residues were coordinated to the metal ions. The first and third aspartate residues in the aspartate-rich motif, D296 and D300, were coordinated to Mg^2+^_A_ and Mg^2+^_C_, which were identical to the BPPS, avian FPP synthase^67^, taxadiene synthase^9^, and *M. spicata* limonene synthase^6^. The second metal-binding region comprised of D440, T444 and E448 of the helix H coordinated to the Mg^2+^_B_. Similar metal ion coordination by the corresponding residues was also observed in trichodiene synthase^71^, 5-*epi*-aristolochene synthase^72^ and taxadiene synthase^9^. The distances between Mg^2+^ cofactor and the corresponding residues were summarized in Table S1. The ideal distance for metal ion coordination was between 2.0 and 2.2 Å, which was more typically observed in higher-resolution structures^73^. It was revealed that the coordination distance with the metal ion for *Pam*Tps1 was within the range of 2.0–2.75 Å, which was longer than what was expected for Mg^2+^ coordination. Shorter metal–ligand distances resulted in tighter first coordination sphere ligands, resulting in less wiggle room in the first coordination sphere, and therefore less deviation from the ideal octahedral geometry^74^. Magnesium has the tightest initial coordination sphere closest to ideal octahedral geometry, with a typical Mg—O distance of around 2.1 Å^74^. Validation of metal-binding sites of *Pam*Tps1 revealed that two of the three metal ions exhibited octahedral geometry, while the third had an outlier geometry (Table 2). The gRMSD measures overall deviation of the observed geometry angle from the ideal geometry angle^75^, and *Pam*Tps1 model showed acceptable gRMSD values for the trinuclear magnesium cluster binding sites. The vacancy calculates percentage of vacant coordination sites for a given geometry^75^. This analysis, however, revealed borderline and outlier vacancy values, which probably explained the longer metal coordination distances between magnesium ion and binding sites as discussed previously (Table S1).

**Table 2:** Metal-binding site geometry analysis.

Metal	Ligand	Geometry	gRMSD	Vacancy
Mg^2+^	O	Octahedral	Acceptable	Borderline
Mg^2+^	O	Square planar	Acceptable	Borderline
Mg^2+^	O	Octahedral	Acceptable	Outlier

In addition to metal coordination interactions, the PPi moiety of GPP and FPP were also predicted to accept hydrogen bonds from R259, R437 and K456 residues (Fig. 4; Table S2). Similarly, this finding was observed in other reported plant terpene synthases where PPi binding was accommodated by hydrogen bonds donated from two arginine and one lysine residues^5,8,72^. The R259 of *Pam*Tps1 derived from the R259DR motif may serve as a proton donor to thermodynamically support the PPi cleavage by protonation after the first reaction step^76–78^. Mutational analysis of this residue showed a loss of catalytic activity suggesting the important role of this arginine residue in restricting the PPi^79^. The R437 derived from the extended second metal binding motif (LR437LADDLGTAPFE) in *Pam*Tps1 was also reported to donate hydrogen bond to the PPi of the substrate as observed with the bornyl diphosphate synthase^5^. The K456 residue of the *Pam*Tps1 that was a part of the conserved lysine residue amongst *Tpsb* terpene synthases was located at the H-α1 loop and hydrogen bonded with the PPi of the substrate. The H-α1 loop lysine residue was also observed to donate hydrogen bond to the PPi in the BPPS crystal structure^5^ and limonene synthase^6^. The coordination of three metal ions and hydrogen bond interactions with basic residues of lysine and/or arginine presumably triggered the ionization of the substrate to yield carbocation intermediates that led to the production of terpenoids^80,81^. The substrate coordination and distance with *Pam*Tps1 residues and Mg^2+^ are summarized in Table S2. Based on the proposed mechanism of 5-*epi*-aristolochene synthase^72^ and *Abies grandis* α-bisabolene synthase^82^, the metal-dependent ionization of the substrate resulted in the generation of a negatively charged PPi that was stabilized by Mg^2+^ ions and three basic residues, and which created a positively charged region that drew the PPi away from the carbocations in the hydrophobic active pocket. Thus, the three Mg^2+^ ions and the three basic residues served as the PPi recognition motif in the active site, allowing proper orientation of the substrate while activating the PPi to initiate ionization and catalysis^8^.

The active site of terpene synthases was also characterized by the presence of several aromatic residues crucial for the stabilization of the carbocation intermediates^5,8,9,79^. The docking results revealed that the non-polar hydrocarbon groups of GPP and FPP were buried in the hydrophobic area of the active site surrounded by aliphatic and aromatic residues (Fig. 4C,D). The C_10_ tail of the GPP formed hydrophobic interactions with W268, Y272, V289, T293, I397, T517 and Y523 residues. Meanwhile the W268, Y272, V289, V292, T293, I397, A398, A399, A402, L436 and Y523 residues participated in non-bonded interactions with the C_15_ group of the FPP. Among the active site residues, the non-polar hydrocarbon group of GPP and FPP were located in the aromatic pair’s area surrounded by residues Y523 of the J-K loop and W268 of the helix C at the bottom of the *Pam*Tps1 active site (Fig. 4A,B). Sequences comparison against other terpene synthases suggested that the W268 was a conserved residue, whereas the position equivalent to Y523 could be occupied by aromatic residues of histidine, phenylalanine or tyrosine as mutation of these residues resulted in catalytically impaired catalyst^6,8,79,83^. According to Brandt et al.^76^, the nature and position of these aromatic amino acid residues at the active site of terpene synthases determined the docking orientation of the intermediate prenyl cation and therefore product specificity. In amorphadiene synthase, the aromatic phenylalanine residue (residue in the same position of Y523 of *Pam*Tps1) was similarly involved in positioning of the FPP substrate in the active site, which subsequently stabilized the carbocation intermediates^84^. A similar observation was also reported by Zhang et al.^85^ with *Nicotiana tabacum* 5-epi-aristolochene synthase (TEAS) that catalyzed the cyclisation of FPP into bicyclic 5-*epi*-aristolochene. Mutational analysis of the aromatic amino acids proved the essential role of these residues in the active site for stabilization of the carbocation intermediates^79,85^. Positioning of GPP and FPP in the *Pam*Tps1 active site surrounded by these aromatic residues suggested that this docking analysis was rational and compatible with other crystal structures of terpene synthases.

### Insights into the *Pam*Tps1 active site pocket

Not all terpene synthases have the ability to use multi-substrate. Steric limitations and configuration of the active site center and the overall protein stability contributed by the tertiary protein structure might rule out the use of multi-substrate^12^. The ability of terpene synthases to catalyze multiple substrates has been reported to be contributed by both size and residues of the active site pockets. In general, the active site pocket is slightly larger than the corresponding substrate and product, and size of the cavity is increasingly deeper and wider for increasingly longer chain products^9,86^. The active site of *Streptomyces clavuligens* linalool/nerolidol synthase (bLinS) has been shown to be large enough to accommodate sesquiterpene, which explained the fact that this enzyme recognized FPP as a substrate^64^. It was predicted that the active site pocket of *Pam*Tps1 was also large enough and unconstrained to accommodate FPP, resulting in nerolidol formation.

**Figure 5:**
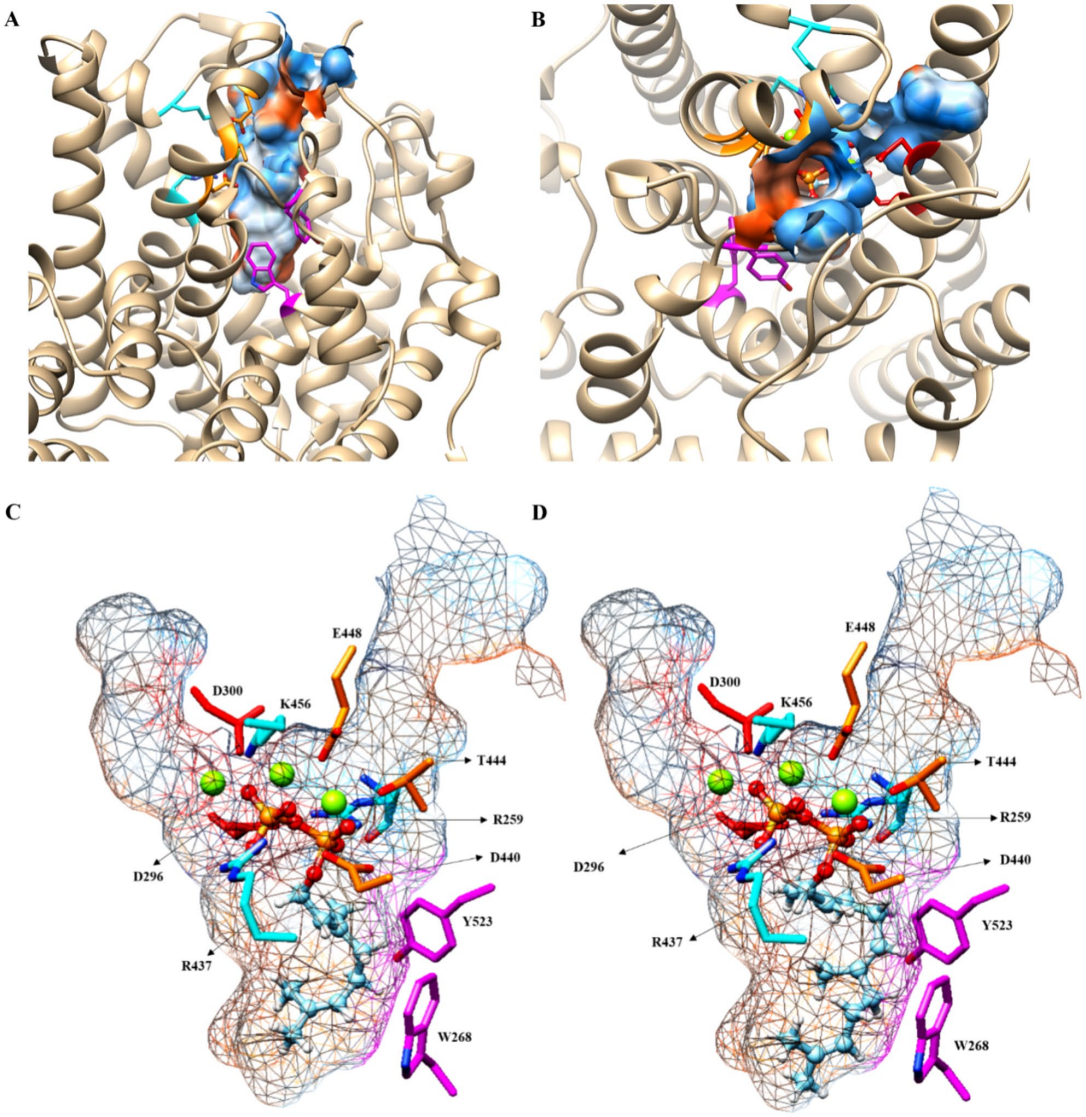
The *Pam*Tps1 active site pocket. The overview of *Pam*Tps1 active site pocket from (**A**) top and (**B**) side views. Docking positions of (**C**) GPP and (**D**) FPP in *Pam*Tps1 active site cavity. The *Pam*Tps1 active site is a deep hydrophobic pocket consisting of C, D, F, G, H and J helices. The red–orange ball and stick chains represent the PPi of substrate, green spheres are the Mg^2+^, the orange side chains are the DTE motifs, the blue side chains are the hydrogen bond donor residues and the magenta side chains represent the aromatic residues.

Using CASTp server and InterProScan analysis, the topographic features of the *Pam*Tps1 active site pocket containing the docked substrate was illustrated in Fig. 5 and amino acids that lined the pocket cavity were also identified (Table 3). Both substrates were appropriately docked in the *Pam*Tps1 active site cavity, thus enlightened the multi-substrate use ability of *Pam*Tps1. The active site cavity of *Pam*Tps1 was a deep hydrophobic pocket with a contour defined by numerous aliphatic and aromatic side chains made of six helices of C, D, F, G, H and J (Table 3; Fig. 5) similarly as described for the BPPS structure^5^. Nine aromatic residues (W268, Y272, Y299, F371, Y378, Y379, F447, Y517 and Y523) outlined the hydrophobic walls of the active site cavity. This result was also supported by structural studies of other plant terpene synthases^5,9,25,72^. It was reported that arginine, phenylalanine, tyrosine, valine, tryptophan and isoleucine were the commonly observed amino acid residues at the catalytic site of the terpene synthases^87^, which was also observed in the *Pam*Tps1 active site. The presence of aromatic residue pairs (Y523 and W268) at the bottom of the active site did not appear to restrict the size of the active site, and the hydrocarbon group of FPP appeared to fit perfectly into the catalytic pocket, which may shed light on the possibility of *Pam*Tps1 accepting FPP as a substrate (Fig. 5). By analogy with the previous characterized enzymes, it was believed that the active site of *Pam*Tps1 was reasonably large and deep enough to accommodate both the GPP and FPP, resulting in the formation of linalool and nerolidol, respectively.

**Table 3:** Amino acid residues involved in the establishment of *Pam*Tps1 active site pocket and catalysis activity predicted using CASTp and InterProScan.

Structure/Motif	Residues	Functions
N-terminal domain (RRx_8_W)	**R**1**R**2**S**3**G**4**N**5**Y**6**Q**7**P**8SAW	Active site lid
A–C loop (RxR)	**R**259DR	Active site lid
		**R**259—Hydrogen bond donor
Helix C	**W**268, **Y**272	Active site pocket
		**W**268—Aromatic residue surrounding non-polar hydrocarbon of substrate
		**W**268, **Y**272—Hydrophobic interaction with PPi
Helix D1	**I**288**V**289, **L**291**V**292**T**293	Active site pocket
		**V**289, **V**292**T**293—Hydrophobic interaction with PPi
Helix D1(DDxxD)	**D**296**D**297V**Y**299**D**300	Active site pocket
		**D**296, **D**300—metal binding motif
Helix F	**F**371, **E**374**A**375, **Y**378**Y**379	Active site pocket
Helix G1	**T**396	Active site pocket
Turn	**I**397**A**398	Active site pocket, hydrophobic interaction with PPi
Helix G2	**A**399, **A**401**A**402	Active site pocket
		**A**399, **A**402—Hydrophobic interaction with PPi
Helix H (L,V)(V,L,A)(N,D)D(L,I,V)x(S,T)xxxE**/**DTE	**L**436**R**437**L**438A**D**440**D**441LG	**F**447**E**448, **E**450- Active site lid
		Active site pocket
		**R**437—Hydrogen bond donor
	**T**444AP**F**447**E**448, **E**450	**D**440, **T**444, **E**448 – metal binding motif
		**L**436—Hydrophobic interaction with PPi
H-α1 loop	**R**451**G**452**D**453**V**454**A**455	Active site lid
	**K**456**A**457, **V**458	**K**456—Hydrogen bond donor
3_10_ helix	**Y**517	Active site pocket, hydrophobic interaction with PPi
J-K loop	**G**520**D**521**G**522**Y**523**G**524**V**525	Active site lid
		**Y**523—Aromatic residue surrounding non-polar hydrocarbon of substrate/Hydrophobic interaction with PPi

Residues from the RRx_8_W motif, RDR motif, H-α1 and J-K loops were observed to act as a catalytic lid that closed the active site entrance upon substrate binding (Table 3). Structural comparison with BPPS showed that the J-K loop of *Pam*Tps1 was longer than the equivalent loop in BPPS. Sugiura et al.^88^ reported that the *Backhousia citriodora* linalool synthase had a long J-K loop and bulky amino acids around the active site that could partially inhibit water access to the active site, leading to the production of (−) linalool and minor amounts of myrcene and (−) limonene. Alignment of amino acids indicated that most Lamiaceae linalool synthases differed from other terpene synthases by a three-amino acid deletion at the –-K loop region, thereby resulting in a more open structure, allowing easier access of water during substrate ionization^25,89^. However, no amino acid deletions at the J-K loop region were observed in the *Pam*Tps1 and it was assumed that the longer J-K loop could lead to the more open structure of the enzyme. The crystal structure of bLinS was also reported to be relatively open, allowing the carbocation intermediate to attack nearby water and led to linalool production^64^. Although the crystal structures *Pam*Tps1’s open and closed active site conformations are not yet available, it is thought that *Pam*Tps1 does not undergo significant conformational changes between open and closed states, as observed with other linalool synthases^25,64^. As a result, the active site was more susceptible to water access, resulting in the premature released of the carbocationic intermediates and the production of acyclic linalool and nerolidol^25,64,89^. Besides that, other conserved motifs considered to be part of the active site were LQLYEASFLL and GTLxEL^63,66^. However, there was no computational evidence that both motifs were involved in the formation of the *Pam*Tps1 active site or in the catalysis reaction, as exhibited by the active site pocket analysis and protein docking studies.

## Conclusion

*Pam*Tps1 was classified as a linalool/nerolidol synthase with the ability to convert GPP and FPP into acyclic linalool and nerolidol, respectively. The catalytic activity of this recombinant synthase was optimal at pH 6.5 and 30 °C in the presence of 20 mM Mg^2+^ as a cofactor, which was within the range of most reported terpene synthases. *Pam*Tps1 catalysis was still stimulated by Mn^2+^ at the optimal concentration of 0.5 mM in place of Mg^2+^, however the catalytic activity was decreased by 2.1 folds. The kinetic properties of *Pam*Tps1 were analyzed using Michaelis–Menten equation, which revealed that it had a higher binding affinity and catalytic efficiency for GPP rather than FPP, as anticipated for a monoterpene synthase located in the plastid where the GPP pool was accessible. The *Pam*Tps1 model structure was successfully constructed from its amino acid sequences using BPPS as a template, and this model will serve as a first glimpse into the structural insights of *Pam*Tps1 catalytic site as a linalool/nerolidol synthase. The *P. amboinicus* linalool/nerolidol synthase exhibited features of a class I terpene synthase fold made up of α-helices architecture that contain the N-terminal domain and a catalytic C-terminal domain. Based on the prior knowledge of the reaction mechanisms of other monoterpene/sesquiterpene synthases, it is hypothesized that a *Pam*Tps1 reaction mechanism begins with the metal-dependent ionization of the PPi moiety of respective GPP or FPP to form a geranyl cation or farnesyl cation. Assisting the metal ions in PPi complexation are the basic residues of R259, R437 and K456 that direct the PPi away from the active site after ionization. The addition of water to the cationic intermediate, followed by deprotonation, resulted in the formation of acyclic terpenoids linalool and nerolidol. The ability of *Pam*Tps1 to use multiple substrates was believed to be due to the enzyme’s active site that was large enough to accommodate larger substrate such as FPP, allowing water capture that caused premature termination and subsequent nerolidol formation. This model will serve as a framework for exploring the roles of active site residues in rational design to exchange the enzyme function between monoterpene and sesquiterpene synthase.

## Materials and methods

### Plant material

The leaves of *P*. *amboinicus* were collected from purchased plants which were maintained at the Faculty of Biotechnology and Biomolecular Sciences, Universiti Putra Malaysia in Selangor, Malaysia (3° 00′ 26.4″ N 101° 42′ 19.3″ E). Plant authentication was performed by a botanist, Dr. Shamsul Khamis, from the School of Environmental Science and Natural Resources, Universiti Kebangsaan Malaysia, Selangor, Malaysia.

### Functional characterization of recombinant *PamTps1*

The *P. amboinicus* linalool/nerolidol synthase (designated as *PamTps1*) (GenBank accession no: MK050501) was previously isolated and functionally characterized by Ashaari et al.^15^. Enzyme assay was conducted in a 100 µl reaction containing assay buffer (10 mM Tris–HCl, pH 7.5, 10% (v/v) glycerol, 1 mM DTT, 0.1 mM NaWO_4_, 0.05 mM NaF), 10 mM MgCl_2_ and 3–5 µg of purified protein. The enzymatic reaction was initiated by addition of 27 µM of GPP or FPP (Sigma Aldrich, USA) and incubated at 30 °C for 30 min. The terpene products released into the headspace of the assay mixture were collected by solid phase micro extraction (SPME) with a 100 µm polydimethylsiloxane (PDMS) coated fiber (Supelco, USA) at 60 °C for 30 min. The adsorbed products were separated through Agilent HP-5MS column (30 m × 250 µm inner diameter × 0.25 µm film thickness) and analyzed using Agilent 7890A gas chromatograph equipped with Agilent 5975C quadrupole mass spectrometer (Agilent Technologies, Santa Clara, USA). The SPME fiber containing the volatile compounds was inserted into GC injection port and thermally desorbed at 250 °C for 15 min using splitless mode with helium as carrier gas at a flow rate of 1 ml/min. The oven temperature was initially maintained at 50 °C and gradually increased to 280 °C at a rate of 10 °C/min for 3 min. The temperature of the ion source and transfer line was set at 220 °C and 280 °C, respectively, and electron impact mass spectra was recorded at 70 eV ionization energy. All assay products were identified by comparison of the mass spectra to the NIST14 library database and by comparing the retention times and mass spectra to the authentic standards of (−) linalool and nerolidol (Sigma Aldrich, USA). Standard calibration curves were constructed using the pure standards with concentrations ranging from 10 to 1000 µg/ml in the same conditions as the assay reactions.

Optimum temperature and pH of *Pam*Tps1 were determined by assaying at various temperatures ranging from 25 to 37 °C and seven pH levels, respectively. The buffer systems used in this study were 2-(N-Morpholino) ethanesulfonic acid (MES) buffer (pH 5.5–6.5) and Tris–HCl buffer (pH 7.0–9.0). Divalent cation preferences and optimum concentrations were determined by assaying at different MgCl_2_ (0.0, 2.0, 4.0, 10.0, 20.0, 50.0, 100, 250 mM) and MnCl_2_ (0.0, 0.1, 0.2, 1.0, 5.0, 10.0 mM) concentrations. The substrate dependence of *Pam*Tps1 was studied by adding GPP or FPP with different concentrations ranging from 0 to 200 µM to the reaction mixture. The kinetic parameters *K*_m_, *V*_max_, *k*_cat_ and *k*_cat_/*V*_max_ values were determined by fitting the data to the Michaelis–Menten equation analyzed using GraphPad Prism8. Extracted total crude proteins from Rosetta 2 (DE3) *E. coli* cells carrying empty pET-32b(+) vector were used as a negative control in place of *Pam*Tps1. One unit (U) of activity was defined as the amount of enzyme required to produce 1 µmole enzymatic product per min per ml under standard conditions. Specific activity was defined as enzyme activity (U) per mg of protein.

### Secondary structure and 3D structure prediction

The motifs and domains were identified using MOTIFinder Search (https://www.genome.jp/tools/motif/), SMART (Simple Modular Architecture Research Tool) (http://smart.embl-heidelberg.de/) and InterProScan^90^. Secondary structure and domain boundary were predicted using PSIPRED Protein Structure Prediction (PSIPRED v3.3)^56^ (http://bioinf.cs.ucl.ac.uk/psipred/) and Protein Domain Prediction (DomPred)^91^ (http://bioinf.cs.ucl.ac.uk/psipred/?dompred), respectively. The three-dimensional protein structure of *Pam*Tps1 model was constructed from the amino acid sequence using automated comparative protein modelling server SWISS-MODEL^92^ (https://swissmodel.expasy.org/) and visualized using UCSF Chimera v 1.13rc^93^. The template for building the 3D structure of *Pam*Tps1 was obtained from the SWISS-MODEL Template Library and the most homologous sequence was considered as a potential template for the homology modeling^92^. The structural superimposition and calculation of the root-mean-square deviations (RMSD) between the model and template were conducted via Chimera using the carbon alpha (Cα) fitting method.

### Validation of the *Pam*Tps1 model

The 3D model was evaluated by SWISS-MODEL’s Global Model Quality Estimation (GMQE) and Qualitative Model Energy Analysis (QMEAN) function. Structural evaluation and stereochemical analysis was conducted with Ramachandran plot using RAMPAGE server^94^ (http://mordred.bioc.cam.ac.uk/~rapper/rampage.php). The model was further subjected to the Structural Analysis and Verification Server v. 5.0 (SAVES) (http://servicesn.mbi.ucla.edu/SAVES/) which included PROCHECK^95^, PROVE (PROtein Volume Evaluation)^96^, ERRAT^97^ and Verify3D analysis^98,99^ to evaluate the reliability of the predicted protein structure. Problematic region of the model was identified using Protein Structure Analysis (ProSA) server (https://prosa.services.came.sbg.ac.at), a tool commonly used to check 3D model protein structures for potential errors^100^.

### Molecular docking

Protein–ligand docking simulation was conducted using the SwissDock server^101^ with the ligand selected from the ZINC database^102^. The docking assays were run using default parameters and the results were viewed via the Chimera software. Hydrogen bond network and distance between ligand and active site residues were also analyzed using Chimera. Distances of the amino acid residues which interacted with Mg^2+^ were also calculated. Identification of amino acids surrounding the active site was conducted by searching for atoms within < 5 Å of the docked ligand. Validation of metal-binding site was conducted using CheckMyMetal server^74^ (https://cmm.minorlab.org/) to assess the geometry of the metal-binding site and the vacancy of the metal.

### Active site pocket analysis

Predictions of the active site pocket and of the amino acid(s) that contributed to the pocket were conducted by applying the CASTp 3.0 server (Computed Atlas of Surface Topography of Proteins)^103^.

### Ethical statement of research involving plants

The *P. amboinicus* that was used in this study was purchased from Petani Kota Nursery located at Dengkil, Selangor, Malaysia (2° 53′ 38.7″ N 101° 45′ 9.0″ E), and it is from cultivated origin. All the methodology and data collection comply with relevant institutional, national and international guidelines and legislation.

## Supplementary Information

Supplementary Tables.

Supplementary Figures.

## Data Availability

Data deposition: the sequences reported in this paper have been deposited in the GenBank database (GenBank Accession No. MK050501).
