# Supplementary material for: Kinetic studies and homology modeling of a dual-substrate linalool/nerolidol synthase from Plectranthus amboinicus
Source: Sci Rep. 2021 Aug 24;11:17094. doi: 10.1038/s41598-021-96524-z (PMC8385045; doi:10.1038/s41598-021-96524-z)
Supplement: Supplementary file 1 — Supplementary Tables. [file 41598_2021_96524_MOESM1_ESM.docx]

**Table S1.** **Coordination and distance of Mg^2+^ with corresponding residues as predicted in *Pam*Tps1 structure**

| **Mg^2+^ no.** | **Residue** | **Distance** |
| --- | --- | --- |
| Mg^2+^_A_ | D296 | 2.74 Å |
|  | D300 | 2.18 Å |
| Mg^2+^_B_ | D440 | 2.37 Å |
|  | T444 | 2.25 Å |
|  | E448 | 2.17 Å |
| Mg^2+^_C_ | D296 | 2.40 Å |
|  | D300 | 2.10 Å |

Table S2. Coordination and distance of GPP and FPP with *Pam*Tps1 residues and Mg^2+^

| **Substrate** | **Docking score** | **Full Fitness** | **Hydrogen bond** | **Atom** | **Residue/Cofactor** | **Distance** |
| --- | --- | --- | --- | --- | --- | --- |
| **GPP** | -106.157 | -2937.4248 | 4 | Oδ3 | R259 | 2.18 Å |
|  |  |  |  | Oδ5  Oδ6 | R437 | 3.10 Å  3.08 Å |
|  |  |  |  | Oδ7 | K456 | 3.24 Å |
|  |  |  |  | Oδ5 | Mg^2+^ _A_ | 2.27 Å |
|  |  |  |  | Oδ2  Oδ7 | Mg^2+^ _B_ | 2.48 Å  2.15 Å |
|  |  |  |  | Oδ5 | Mg^2+^ _C_ | 1.94 Å |
| **FPP** | -102.248 | -2917.827 | 4 | Oδ3 | R259 | 2.16 Å |
|  |  |  |  | Oδ5  Oδ6 | R437 | 3.07 Å  3.07 Å |
|  |  |  |  | Oδ6 | K456 | 3.07 Å |
|  |  |  |  | Oδ4  Oδ7 | Mg^2+^ _A_ | 2.24 Å  2.30 Å |
|  |  |  |  | Oδ2  Oδ6 | Mg^2+^ _B_ | 2.14 Å  2.43 Å |
|  |  |  |  | Oδ7 | Mg^2+^ _C_ | 1.92 Å |

**Table S3:** **Comparison of *Pam*Tps1 3D model structure evaluation with BPPS (1N24) template**

| **Validation** | **Analysis** | **Threshold values** | **BPPS (1N24)** | ***Pam*Tps1** |
| --- | --- | --- | --- | --- |
| SWISS-MODEL | Sequence identity |  | 67.04 % | |
|  | GMQE | 0-1 | 0.82 | |
|  | QMEAN | 0 | -1.32 | |
| Chimera | RMSD | Superimposition | 0.203 Å | |
| PROCHECK | Ramachandran plot | Favored region | 92.8 % | 92.8 % |
|  |  | Additional allowed | 7.0 % | 6.6 % |
|  |  | Generously allowed | 0.2 % | 0.2 % |
|  |  | Disallowed region | 0.0 % | 0.4 % |
| PROVE | Z-score mean |  | n/a | 0.487 |
|  | Z-score RMS | 1-3.9 Å | n/a | 1.421 |
|  | Outliers |  | 2.8 % | 4.7 % |
| ERRAT | Quality factor | ≥ 95 % | 100 % | 95.88 % |
| Verify3D | 3D/1D profile | ≥ 80 | 73.14 % | 95.73 % |
| ProSA | Z-score | Close to template value | -10.92 | - 12 |
