# Supplementary material for: Kinetic studies and homology modeling of a dual-substrate linalool/nerolidol synthase from Plectranthus amboinicus
Source: Sci Rep. 2021 Aug 24;11:17094. doi: 10.1038/s41598-021-96524-z (PMC8385045; doi:10.1038/s41598-021-96524-z)
Supplement: Supplementary file 2 — Supplementary Figures. [file 41598_2021_96524_MOESM2_ESM.docx]

**Supplementary figure**


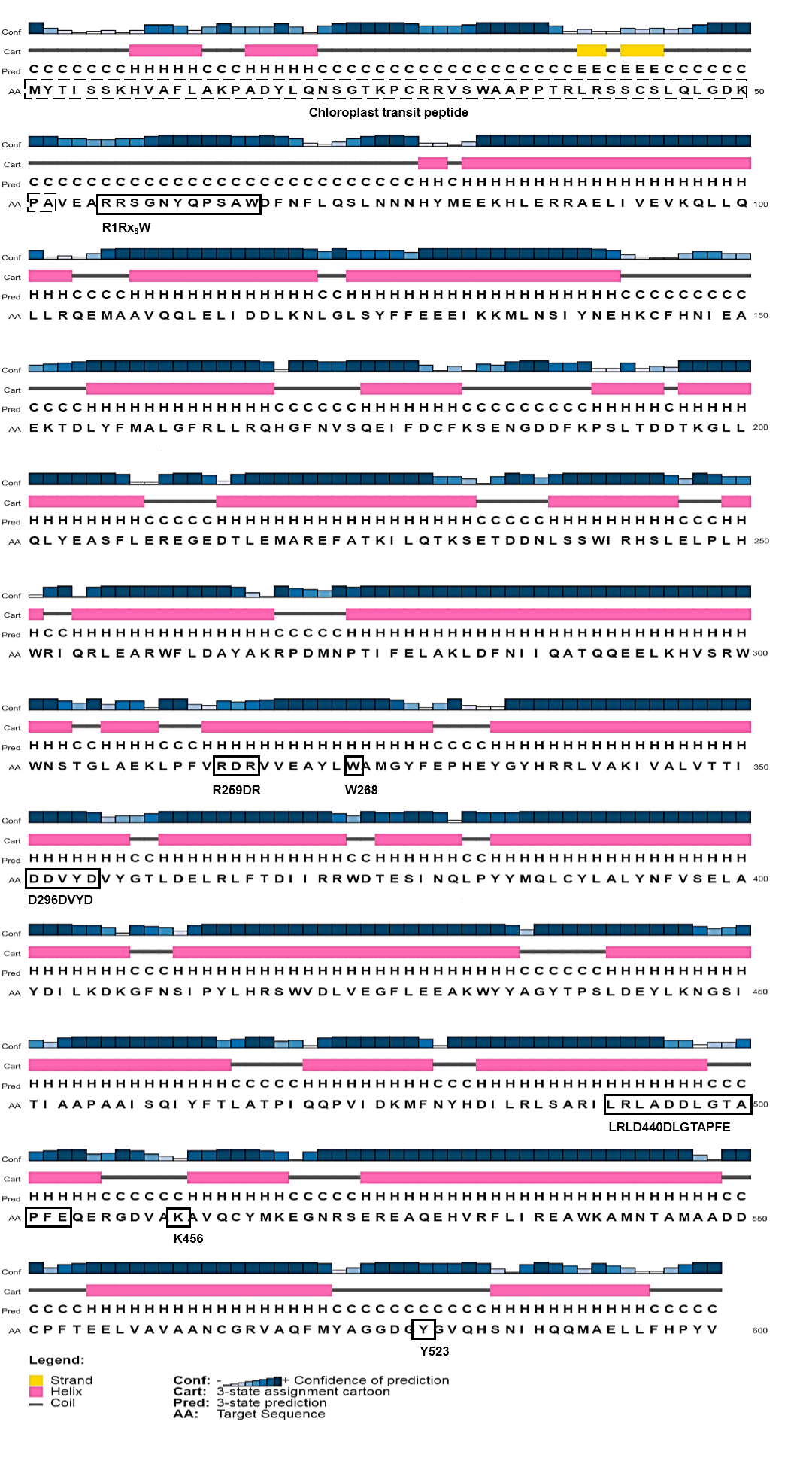


**Fig. S1**: **Predicted secondary structure of *Pam*Tps1 using PSIPRED server**. The *Pam*Tps1 consists of 24 α-helices connecting by coils. Chloroplast transit peptide and all conserved motifs of terpene synthases including RRx_8_W, RxR, DDxxD and NSE/DTE were labelled (Table 2).


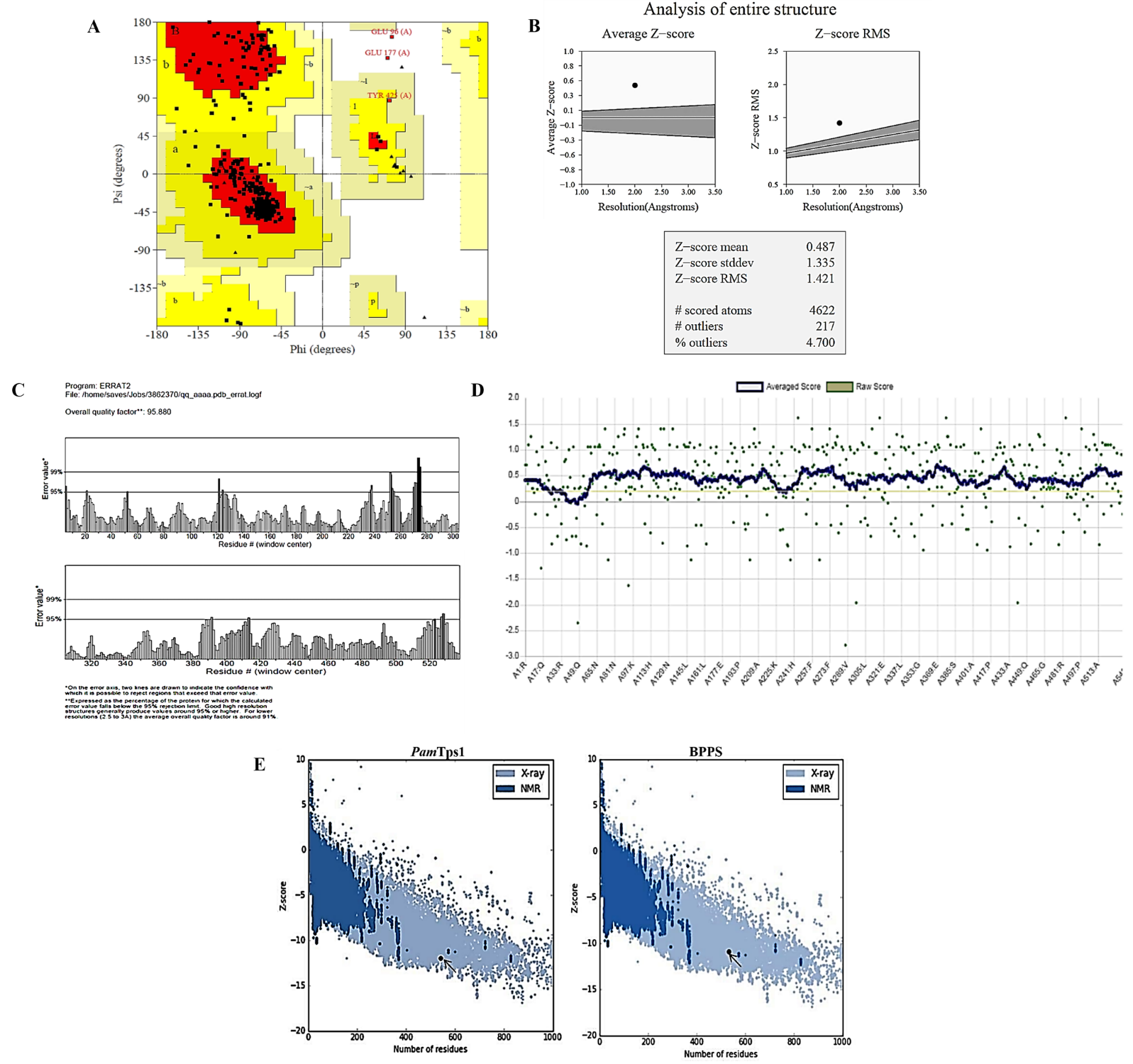


**Fig. S2**: **Validation of *Pam*Tps1 model**. (A) Ramachandran plot analysis indicating 92.8 % of *Pam*Tps1 residues fell in the most favored regions (red), 6.6 % of residues in the additional allowed regions (yellow), 0.2 % of residues in the generously allowed regions (light yellow) and only 0.4 % of residues within the disallowed regions (white) (B) PROVE. The Z-score and Z-score RMS for *Pam*Tps1 were 0.487 and 1.421, respectively (C) Overall quality of the model evaluated by the ERRAT program scored 95.88 % (D) The Verify3D result of *Pam*Tps1 model with 95.73 % of the residues had an average 3D-1D score ≥ 0.2 (E) The Z-score value of ProSA plot (displayed as black dot) for protein model and the template. The Z-score of the *Pam*Tps1 model of -12 was closed to the value of the BPPS template (-10.92).
